# Effect of type of farming practices on the soil carbon sequestration and yield of some crops

**DOI:** 10.1038/s41598-026-35230-0

**Published:** 2026-01-29

**Authors:** El-Sayed Khater, Adel Bahnasawy, Ramy Hamouda, Amr Sabahy, Wael Abbas, Osama Morsy, Mahmoud El-Habbaq

**Affiliations:** 1 Agricultural and Biosystems Engineering Department, Faculty of Agriculture, Benha University, P.O. Box 13736, Moshtohor, Toukh, Kalubia Egypt; 2 Faculty of Organic Agriculture, Heliopolis University, P.O. Box 11785, Cairo, Egypt; 3 Institute of Agricultural Engineering Research, Agriculture Research Center, Doki, Giza, Egypt; 4 Basic and Applied Science Department, College of Engineering and Technology, Arab Academy for Science and Technology and Maritime Transport (AASTMT), P.O. Box 2033, Cairo, Egypt; 5 Agricultural Ecоnоmics Department, Faculty of Agriculture, Benha University, P.O. Box 13736, Moshtohor, Toukh, Kalubia Egypt

**Keywords:** Conventional, Organic, Biodynamic, Crop yield, Soil carbon sequestration, CO_2_ emission, Cost, Ecology, Environmental sciences, Engineering

## Abstract

Soil carbon sequestration is a long-time storage of carbon in soil which represents 70% of the carbon in land. Therefore, the main aim of this study is to investigate the effect of the agricultural practice systems on the soil carbon sequestration and properties, productivity, water consumption, soil carbon sequestration, CO_2_ emission and cost of some agricultural crops. To achieve that, different farming systems (conventional, organic and biodynamic) and four crops (maize, tomato, faba bean and potato) were used during 5 agricultural years. The obtained results indicated that, the agricultural practices for different farming systems enhanced the soil properties. Biodynamic practice farming causes reduction in bulk density, which it increase the water holding capacity of the soil which in turn decreased the water consumption by plants. Regarding the chemical properties of the soil, biodynamic and organic farming improved the chemical characteristics such as pH, EC, N, P and K compared to the conventional practice farming. Yield values of both biodynamic and organic farming system were higher than that of the traditional farming system. The amount of soil carbon sequestration ranged from 1980.17 to 4782.82, 2505.89 to 6132.38 and 1581.07 to 5986.25 kg ha^− 1^ for conventional, organic and biodynamic systems, respectively. The amount of CO_2_ emission reduction for organic and biodynamic systems was higher than those of conventional system during experimental period. The highest value of carbon profit (13,071.60 Egyptian pound per hectare (EGP ha^− 1^), $=48.48EGP) was found with the biodynamic system. The highest values of total net profit were 25,046.64, 67,463.04, 44175.84 and 94,674.24 EGP ha^− 1^ for maize, tomato, faba bean and potato crops, respectively, were found with the organic farming system after 5 agricultural years.

## Introduction

Sustainable agricultural system is the system which aims to ensure food security while saving natural resources need to be used on a broad scale. Recently, organic agricultural systems have drawn much attention as alternative ways to produce food and ensure security in turns of environmental sustainability^1–3^. To increase food production to overcome the needs of increasing population has driven the intensification of agricultural practice within the last century, with severe effects of environmental health. As a result, nutrient cycling, greenhouse gas emissions and biodiversity loss have been crossed as key conditions, also due to agricultural practices^4^.

Soil quality is considered important factor affecting ecosystem, where by controlling nutrient and water cycles and providing habitat for soil biodiversity^5,6^. Soil processes are very complex in nature, and the changes in soil quality should be monitoring day-to-day by regulating farming operation and policy decisions^5^. Soil organic carbon (SOC) is considered one of the most important soil quality indicators, followed by soil pH and available phosphorus^7^.

The SOC content has a direct and indirect impact on biological, chemical, and physical soil properties. Increasing SOC contents can partly substitute anthropogenic CO_2_ emissions^8–10^. However, in the history of agriculture, SOC contents have mostly been diminished rather than removed^11^. Nowaday, SOC contents decreased in the intensified agricultural system^12^. This is particularly true when grassland is converted to arable farming^13^. Moreover, SOC losses may increase in the coming decades due to warming that triggers enhanced SOC mineralization in agricultural soils. Wiesmeier et al.^14^ have postulated an additional need for organic matter inputs to agricultural soils in order to stabilize SOC pools in Central European soils. Consequently, there is an urgent need for agricultural practices to counteract SOC losses and build additional SOC^15^.

In organic farming system, the crop is grown with no chemical usage. Besides, it maintains biodiversity and reduces the anthropogenic footprint on soil, air, water, wildlife, and especially on farming communities. Organic farming is one of the best ways not only to reduce the deterioration of water quality but also to decrease food toxicity. Fields that have been continuously managed organically for years have lower numbers of pests, which has been attributed to increased biodiversity and abundance of multiple trophic interactions as well as changes in plant metabolites^16^.

The DOK experiment (bioDynamic, bioOrganic, Konventionell (traditional German)) was conducted in 1978 in Therwil (CH). After 42 years, SOC contents increased in biodynamic (BIODYN) and to a lesser extent also in bioorganic (BIORG). Conventional (CONFYM) showed 1.4 livestock unit per hectare stable SOC contents, while the manure-enriched systems lost 0.7 livestock unit per hectare and purely mineral fertilized system (CONMIN). The highest SOC loss was in unfertilized control (NOFERT). The improvement of biological soil quality under organic management and especially biodynamic management highlights the close link between soil biology and SOC changes. Manure recycling at a level of 1.4 livestock unit per hectare allows for the maintenance of SOC levels and composting, as done in BIODYN 1.4, helps increase SOC levels and improve biological soil quality^3^.

In the last century, agricultural intensification secured the burgeoning demand for food by a growing population against of soil and environmental health^17^. Consequently, societal concerns stimulated the development of sustainable agricultural practices in the last decades^18^, and today organic farming systems have gained momentum in food production worldwide^19^. Since, using the mineral fertilizers for plant nutrition and chemical plant protection strategies are prohibited, organic farming systems often result in lower yields, but present benefits for biodiversity, nutrient cycling and environmental quality^20^. Organic farming systems improve the soil quality indicators such as soil organic carbon contents, microbial biomass and soil respiration^3^. The addition of organic inputs enhances soil microbial community structure in organic and conventional farming systems in temperate climates^21^.

The provision of plant-available nitrogen to secure crop production presents is the important due to the emission of mineral nitrogen fertilizers in organic farming. Traditional agricultural practices mitigate this challenge by applying mineral nitrogen sources; globally, *>* 80 million tons of nitrogen fertilizers are applied to soils annually^22^. Negative environmental effects result in due to induce the loss of nitrogen from agricultural systems includes the emission of greenhouse gases, such as nitrous oxide, and the eutrophication of distant ecosystems and surface waters^23^. Consequently, managing soil nitrogen cycling is crucial for the development of sustainable agricultural production^24,25^. Denitrification is the stepwise reduction of nitrate under anoxic conditions to dinitrogen through the obligatory intermediates nitrite, nitric oxide and nitrous oxide. The last step of denitrification, the conversion of nitrous oxide to dinitrogen, is encoded by the functional gene*nosz*^26^ and is of special environmental relevance due to its mitigation potential for agricultural N_2_O emissions^27,28^.

The traditional agricultural practices result in many problems such as soil deterioration and excessive water consumption in addition to increasing the CO_2_ emission. Changing the agricultural practice system to organic and biodynamics practices could help in reducing the problems resulting from the traditional practice, therefore, the main aim of this study is to investigate the effect of the agricultural practice systems on the soil carbon sequestration and properties, yield, water consumption, CO_2_ emission and cost of some agricultural crops.

## Materials and methods

The experiment was conducted out at a privet farm at Belbeis, Al-Sharqia Governorate, Egypt (30.421 Latitude and 31.635 Longitude), during the period of 2017–2022. By the permission of bellies company regulation and rules, the experiment was conducted in the land that devoted for carrying out the field experiments. The ambient air temperature ranged from 13.6 to 31.9 °C, the relative humidity ranged from 43.0 to 57.1% and solar radiation ranged from 342.5 to 964.1 kJ m^− 2^ day^− 1^.

### Experimental setup

The field experiments were consisted of 27 plots, plot size is 20 × 12 m and the total area of plot is 240 m^2^. Five years crop rotation with maize and tomato in summer season and faba bean and potato in winter season in three replicates. The soil texture in this trial was sandy soil. The physical, mechanical and chemical analysis of the experimental soil is shown in Table 1.

**Table 1:** The physical, mechanical and chemical properties of the soil used at the beginning of the experiment.

Physical properties					Mechanical properties									
Bulk density, kg m^−3^	Soil porosity, %	Field Capacity, %	Wetting point, %	Water holding capacity, %	Sand	Silt	Clay	Texture						
1654	38	9.10	4.90	4.20	88.0	9.5	2.5	Sand						

Chemical properties														
pH	EC, dS m^−1^	Anions, meq L^−1^			Cations, meq L^−1^				SOM, %	SOC, %	C/N ratio	Total N, %	Total P, %	Total K, %
		HCO^-3^	CL^-1^	SO^-4^	Ca^++^	Mg^++^	Na^+^	K^+^						
7.58	1.49	3.30	15.67	10.57	9.21	4.92	14.66	0.84	0.31	0.17	4.25	0.04	0.09	0.05

Drip irrigation system was used in this study, which consists of control head (centrifugal pump (Model PEDROLLO—Flow Rate 30 m^3^ h^− 1^—Head 48 m—Power 4.0 kW, Italy), pressure regulator, pressure gauges, flow meter and filtration unit), PVC main, sub-main, secondary and manifold lines with 110 and 90, 63 and 50 mm diameters. Emitters (GR) used in this work were installed in lateral lines of polyethene (PE) with a diameter of 16 mm, distance of 30 cm and emitter’s discharge of 4 L h^− 1^. The manifold line was equipped with a valve, a water meter and a pressure gauge, located at the beginning of the laterals. Water consumptive use (mm day^− 1^) was calculated and applied according to the climate data using the Penman–Monteith method described by FAO^29^, and according to local weather station data, which located in Belbeis.

### Cultivated crops

Different crops were cultivated in the experimental area of trial under the long-term effects of farming systems for 5 years, two crops each agricultural season: in the winter season, faba bean (Sakha-3 variety) and potato (Cara variety) were grown, while in the summer season, maize (Hytech 2055 variety), and tomato (Beto 86 variety) were grown. The source of crops were the faculty of Agricultural station, moshtohor, benha University, Egypt. Fertilizer requirements of different crops were applied as recommended by Agronomy Research Institute, ARC, Ministry of Agriculture and Land Reclamation.

### Experimental design

**Fig. 1:**
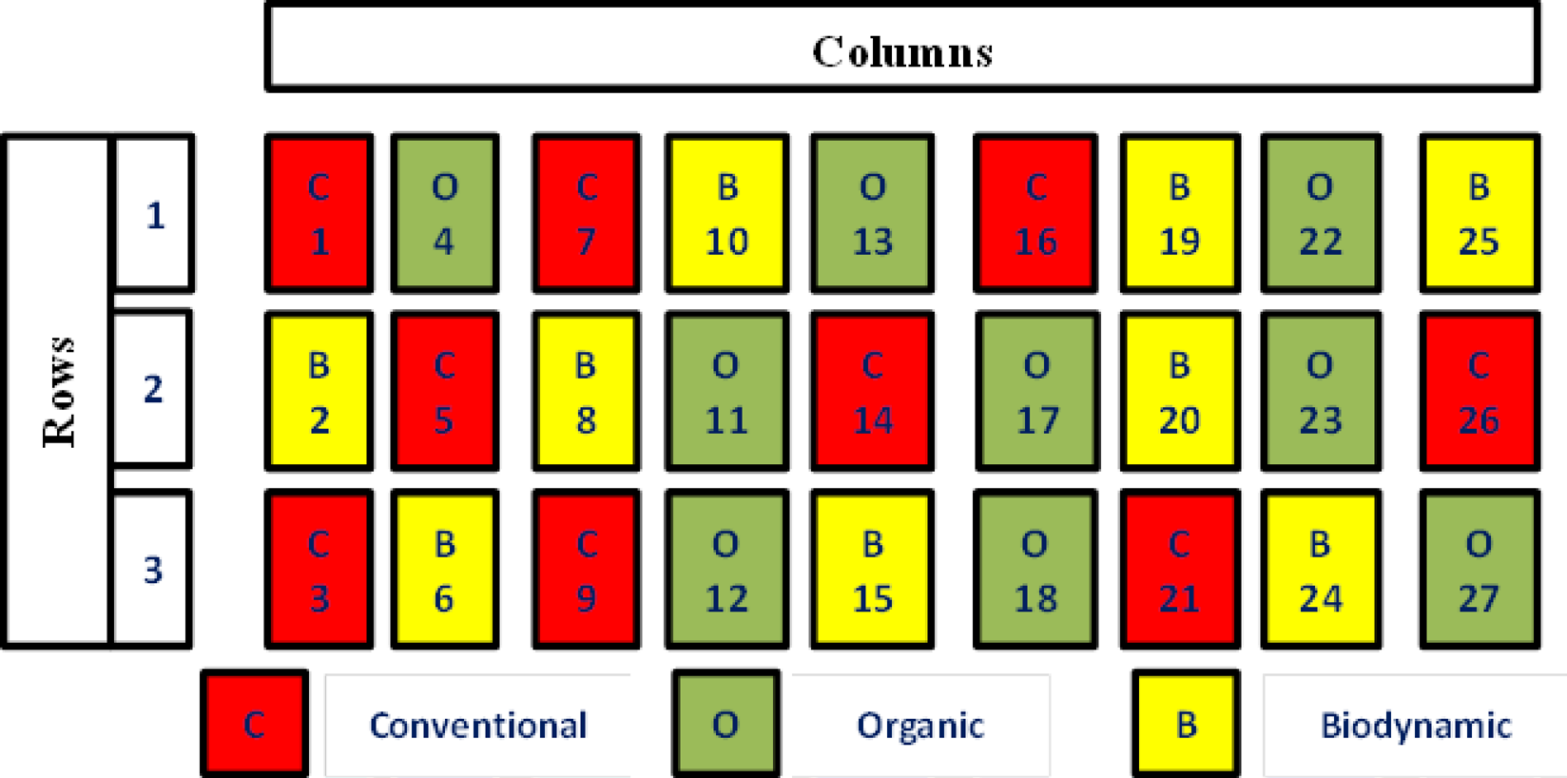
Experimental design for different farming systems.

The treatments were arranged in a completely randomized block design in three replications and three farming systems (conventional, organic and biodynamic) and four crops (maize, tomato, faba bean and potato). Figure 1 shows the experimental design.

### Farming systems

#### Soil carbon sequestration (SCS)

Soil carbon sequestration is defined as carbon stored in soil.

#### Conventional farming system

In this system, the chemicals were used different crops during experimental period (5 years) are listed in Table 2. These include: (Ammonium Nitrate (34 N- 0P-0 K), superphosphate 15.5 P, Calcium Nitrate 15 Ca and Potassium 48 K. All these fertilizers under study were placed in separate tanks and sprayed on the different plots using a 20-liter hand sprayer at the specified time with the recommended dose.

**Table 2:** Chemicals used in conventional farming system for different crops.

Fertilizer	Typology	Maize	Tomato	Faba bean	Potato
NH_4_NO_3_-equivalent, kg ha^− 1^	Fertilizer	283.2	372	120	264
P-equivalent, kg ha^− 1^	Fertilizer	79.2	180	72	192
K-equivalent, kg ha^− 1^	Fertilizer	72	108	72	192
NPK(12-12.36 + TE), kg ha^− 1^	Fertilizer	12	24	0	36
Ammonia nitrate 20.6%, kg ha^− 1^	Fertilizer	120	144	24	144
Urea fertilizer, kg ha^− 1^	Fertilizer	144	72	0	120
Magnesium sulphate, kg ha^− 1^	Fertilizer	84	79.2	24	144
Somethion 50% E.C, L ha^− 1^	Insecticide	3.6	4.8	0	4.8
Chlorzan 48% E.C, L ha^− 1^	Insecticide	0	3.6	0	4.8
Chemidazed 50%, L ha^− 1^	Insecticide	4.8	4.8	2.4	4.8
Brims 2.5% FS, L ha^− 1^	Insecticide	2.4	4.8	4.8	2.4

Organic farming system

The fertilizers used in organic farming system for different crops during experimental period (5 years) are listed in Table 3. Compost was added during soil preparation in the organic farming systems. The component analyses of the used compost in the system are shown in Table 4.

**Table 3:** Chemicals used in organic farming system for different crops.

Fertilizer	Typology	Maize	Tomato	Faba bean	Potato
Compost, ton ha^− 1^	Fertilizer	24	28.8	12	36
Compost tea, L ha^− 1^	Fertilizer	288	528	132	744
Biogas manure, m^3^ ha^− 1^	Fertilizer	19.2	24	7.2	52.8
Natural rock, kg ha^− 1^	Fertilizer	288	432	72	432
Bio-N, L ha^− 1^	Fertilizer	4.8	4.8	2.4	7.2
Bio-P, L ha^− 1^	Fertilizer	4.8	7.2	2.4	4.8
Bio-K, L ha^− 1^	Fertilizer	4.8	4.8	2.4	4.8
Kelpak, L ha^− 1^	Insecticide	2.4	2.4	0	2.4
Biological agents, L ha^− 1^	Fungicide	2.4	2.4	2.4	4.8
Humic acids, L ha^− 1^	Fertilizer	7.2	7.2	0	7.2
Sorel Microny, L ha^− 1^	Fungicide	2.4	4.8	0	2.4
Plant Guard, L ha^− 1^	Fungicide	2.4	2.4	0	4.8
Nembercidine, L ha^− 1^	Fungicide	4.8	4.8	2.4	7.2

**Table 4:** Properties of the compost used in organic farming system.

Properties	Value
Moisture content, %	25
pH	7.42
EC, dS m^− 1^	7.77
Density, kg m^− 3^	650
Total nitrogen, %	1.26
Organic matter, %	35.91
Organic carbon, %	20.83
Total potassium, %	0.80
Total phosphorus, %	1.05
C/N ratio	16:1
Ash, %	64.09

Biodynamic farming system

The fertilizers used in this system for different crops during experimental period (5 years) are shown in Table 3. Compost was added during soil preparation in the biodynamic farming systems. Regarding the use of biodynamic farming system, the same practices were implemented in the organic fertilization system, in addition to the use of additional treatments such as spraying liquid horn manure in the amount of 360 g ha^− 1^ one time after planting also, the silica was added at the rate of 4.8 g ha^− 1^ three times per season.

### Measurements

#### Soil physical and chemical properties

Soil samples were taken from the experimental area using sampling auger at 30 cm depth in different places. The soil bulk density (BD) of soil is measured by weighing a pre-determined volume by using the following equation:

1 $$BD = \frac{{mass\;{\mathrm{of}}\;{\mathrm{soil}}}}{{Bulk\;{\mathrm{volume}}\;{\mathrm{of}}\;{\mathrm{soil}}}} \times 100$$

Soil water holding capacity (WHC) was determined by weighing a wet sample (W_*i*_) and placing it in a beaker for 1–2 days using distilled water. Excess water was drained through Whatman #2 filter paper, and the saturated sample was weighed again (*W*_*s*_). The amount of water retained in the sample was calculated using the following equation from Ahn et al.^30^:

2 $$WHC = \frac{{\{ (W_{S} - W_{i} ) + MC \times W_{i} \} }}{{\{ (1 - MC) \times W_{i} \} }} \times 100$$

where: WHC is the water holding capacity, %. *W*_*i*_ is the weight of sample before drying, g. *W*_*s*_ is the weight of sample after drying, g. MC is the initial moisture content of the sample, decimal.

Electrical conductivity and pH were measured in a 1:5 (*v*/*v*) material/water extract using a glass electrode. Organic carbon (OC) was determined by using the dry combustion method at 540 °C for 4 h, as specified by Abad et al.^31^. Organic matter was measured by combustion at 550 °C for 8 h according to TMECC^32^, and total nitrogen (TN) was measured by Kjeldahl digestion (model VAPODEST; range 0.1 mg to 200 g N; Germany) according to Bremmer and Mulvaney^33^. Potassium (K) content was determined by atomic absorption (model EMI9783B; range of 190–930 nm; USA), and phosphorus (P) content was determined calorimetrically method^34^.

#### Water consumption

The amount of irrigation water requirements under different farming systems (conventional, organic and biodynamic) for different crops was determined by using the following equation according to Morad et al.^35^:

3 $$IWR = \frac{{\left( {\left[ {\left( {\theta _{{FC}} - \theta _{v} } \right) \times d} \right] + L_{f} } \right)}}{{E_{s} }}$$

where: IWR is the total amount irrigation water requirements, mm. θ_FC_ is the soil moisture content at field capacity, %. θ_v_ is the soil moisture content before irrigation, %. d is the soil depth, mm. L_*f*_ is the leaching factor, 20% for drip irrigation system according to Ayers and Westcot^36^.

Es is the drip irrigation system efficiency, 85%.

#### Crop yield

The yield of the different crops was determined (ton ha^− 1^) to study under the different farming systems. Three crop samples were taken randomly from the experiment area from each treatment.

#### Water use efficiency (WUE)

Water use efficiency (kg m^− 3^) was determined by used the following equation according to Pene and Edi^37^:

4 $$WUE = \frac{{Cropyield}}{{Amount\;of\;water\;consumption}}$$

####  Cost

Cost analysis was carried out to calculate the optimum economic conditions under different farming systems as follow:

- The total costs were calculated taking into account the costs of all farming operations for soil preparation, fertilization, cultivation cost, pesticide cost, labor cost, energy consumption and total irrigation cost. Table 5 shows the inputs of cost components for three farming systems (conventional, organic and biodynamic) and different crops (maize, tomato, faba bean and potato) during the 5 years farming crops.
  **Table 5:** Inputs of costs calculations.

Item	Crop	Farming System								
		Conventional			Organic			Biodynamic		
		1st year	3rd year	5th year	1st year	3rd year	5th year	1st year	3rd year	5th year
		Cost, LE ha^− 1^								
Fertilizers	Maize	1872	2040	2928	1800	2040	2640	1800	2040	2640
	Tomato	2772	3048	4320	1800	2040	2640	1800	2040	2640
	Faba bean	1368	1500	2124	1800	2040	2640	1800	2040	2640
	Potato	3480	3816	5400	1800	2040	2640	1800	2040	2640
Energy	Maize	432	624	1008	192	504	748.8	240	624	748.8
	Tomato	408	648	744	324	324	600	264	264	600
	Faba bean	72	516	756	48	264	552	48	312	552
	Potato	732	1632	2040	504	1008	1488	504	1008	1488
Water consumption	Maize	3355	5014	7843	2830	3881	5393	2657	3881	5064
	Tomato	4450	5242	8102	3758	4872	6883	3684	4788	5784
	Faba bean	1747	2863	4637	1426	1898	3113	1457	1908	2899
	Potato	4450	8040	11,794	3758	5400	7812	3684	5352	7685
Labor	Maize	1800	1920	2160	2280	2880	4320	2760	3552	5280
	Tomato	3120	3600	4320	3840	4752	6000	4200	4752	7200
	Faba bean	840	1584	1920	2400	2592	2640	2760	3168	3600
	Potato	749	1584	2640	1066	2304	3840	1277	2736	4800
Pesticide	Maize	840	3240	6600	420	418	3648	360	420	3960
	Tomato	2760	4512	6840	420	768	6360	360	768	5880
	Faba bean	864	3480	5568	288	5040	6240	288	5040	6682
	Potato	3240	5052	5052	4320	5760	5760	4320	5760	5760
Seeds	Maize	624	768	1320	624	768	1320	624	768	1320
	Tomato	28,800	38,400	40,800	28,800	38,400	40,800	28,800	38,400	40,800
	Faba bean	864	1008	1560	864	1008	1560	864	1008	1560
	Potato	6240	33,600	50,400	6240	33,600	50,400	6240	33,600	50,400
- Total return was calculated by using the following equation:

5 $$TR = {\mathrm{Crop}}\;{\text{ price}} \times {\mathrm{Yield}}$$

where: TR is the total return, EGP ha^− 1^.

Although there is an increase in the sale price of final organic and biological products by 20 and 25%, respectively, compared to the conventional product according to Ankamah-Yeboah et al.^38^. In this current study, the estimation was performed at the same rate to illustrate the effect of soil carbon sequestration and its relationship to yield in economic terms.

- Net return was calculated by using the following equation:
  6 $$NR = TR - TC$$
  where: NR is the net return, EGP ha^− 1^. TC is the Total costs, EGP ha^− 1^.

- Profit analysis for mitigation of CO_2_ emission was calculated using the following equation:
  7 $$P_{{co_{2} }} = Am_{{co_{2} }} \times Mp$$

where: $P_{{co_{2} }}$ is the profit analysis for mitigation of CO_2_ emission, EGP ha^− 1^. $AM_{{co_{2} }}$ is the amount of mitigation of CO_2_ emission, ton ha^− 1^. Mp is the market prices of CO_2_ offsets, ton ha^− 1^.

The carbon market price per ton was determined according to the Carbon Footprint Center (CFC), Heliopolis University, Egypt, on the basis of the average selling price from 2017/2018 to 2021/2022. The amount of mitigation of CO_2_ emission was determined by using the following equation according to Shin et al.^39^:

8 $$Am_{{{\mathrm{CO}}_{2} }} = {\mathrm{SCS}} \times {\mathrm{CF}}$$

Where: SCS is the soil carbon sequestration, ton ha^− 1^. CF is the conversion factor of CO_2_ emission from carbon (1 kg C = 3.664 kg CO_2_).

- Total profit was calculated as follows:

10 $$TP = NR \times P_{{{\mathrm{CO}}_{{\mathrm{2}}} }}$$

All experimental protocols were approved by Benha University research committee and all methods used in this study were carried out according to the International, National and Benha University guidelines regulations. This work is approved by the ethic committee at Benha University.

### Statistical analysis

The data were subjected to analysis using statistical package SPSS version 21 in which one way ANOVA and Duncan Multiple Range Test (DMRT) were performed at significance level of (*p* < 0.05) at 95% confidence limit to know the significant differences between the treatment means for different parameters.

## Results and discussion

### Soil physical and chemical properties

Table 6 shows the soil properties (physical and chemical) for different farming systems (conventional, organic and biodynamic) during experimental period. The results indicate that the bulk density of the soil changed with the type of farming practice, where, biodynamic system had a lower bulk density compared to both conventional and organic farming systems during experimental period. It could be seen that the average bulk density of soil was 1566.62, 1483.86 and 1483.64 kg m^− 3^ for conventional, organic and biodynamic systems, respectively compared to 1654.32 kg m^− 3^ soil bulk density before the agricultural practices. The results explained that the use of compost with biodynamic additives during the experimental period enhanced soil properties compared to other farming systems. Also, the agricultural practices for different farming systems enhanced the soil bulk density. The soil bulk density was significantly decreased from 1610.20 to 1502.30, 1524.40 to 1426.00 and 1530.60 to 1422.00 kg m^− 3^ with increasing experimental period from 1 to 5 years for conventional, organic and biodynamic systems.

**Table 6:** Soil physical and chemical properties for different farming systems during experimental period.

Parameter	Before	Farming systems								
		Conventional			Organic			Biodynamic		
		1st year	3rd year	5th year	1st year	3rd year	5th year	1st year	3rd year	5th year
Bulk density, kg m^− 3^	1654.32^e^	1610.20^d^	1587.35^d^	1502.30^b^	1524.40^b^	1501.20^b^	1426.00^a^	1530.60^bc^	1498.33^b^	1422.00^a^
Water holding capacity, %	4.20^a^	4.35^ab^	4.42^b^	4.69^c^	4.98^d^	5.25^e^	5.36^e^	5.20^e^	5.43^ef^	5.71^g^
pH	7.6^ab^	7.8^b^	8.1^c^	8.3^c^	7.2^a^	7.4^a^	7.4^a^	7.3^a^	7.4^a^	7.4^a^
EC, dS m^− 1^	0.45^a^	1.10^c^	1.40^d^	1.51^d^	0.91^b^	1.20^cd^	1.14^c^	0.92^b^	1.04^c^	1.14^c^
Organic matter, %	0.30	0.21	0.27	0.18	0.51	0.64	0.71	0.53	0.70	0.72
Organic carbon, %	0.17	0.12	0.16	0.10	0.29	0.37	0.41	0.31	0.41	0.42
C/N ratio	2.00^d^	1.35^b^	1.12^b^	0.62^a^	3.00e	2.96^e^	2.02^e^	3.00e	1.93^d^	1.46^bc^
Total nitrogen, %	0. 0850	0.095	0.140	0.168	0.110	0.189	0.204	0.110	0.210	0.287
Total phosphorus, %	0.0854	0.14	0.452	0.324	0.130	0.314	0.245	0.120	0.245	0.180
Total potassium, %	0. 052	0.087	0.017	0.179	0.160	0.240	0.324	0.170	0.230	]\0.312

Means on the same row with different superscripts are significantly different (*p* < 0.05).

Regarding the water holding capacity (WHC), the results indicate that the WHC of soil for biodynamic system was higher than those of organic and conventional systems during experimental period. The average of soil water holding capacities were 4.49, 5.97 and 5.45% for conventional, organic and biodynamic systems, respectively compared to 4.20% soil water holding capacity before the agricultural practices. Also, the soil water holding capacity was significantly increased from 4.35 to 4.69, 4.98 to 5.36 and 5.20 to 5.71% with increasing experimental period from 1 to 5 years for conventional, organic and biodynamic systems, respectively.

Soil pH ranged from 7.8 to 8.3, 7.2 to 7.4 and 7.3 to 7.4 for conventional, organic and biodynamic systems, respectively. Use of chemical fertilizers (conventional system) increased the value of soil pH (8.3) compared to the other treatments under study. While, the use of organic and biodynamic systems lowered the value of soil pH. These results agreed with those obtained by Lori et al.^40^ whose found that the organic farming system gave the best soil quality compared to conventional farming system. The results indicate that the highest value of soil EC (1.51 dS m^− 1^) was found with the conventional farming system, while, the lowest value of soil EC (0.92 dS m^− 1^). The average of soil organic matter and organic carbon were 0.22, 0.62 and 0.65 and 0.13, 0.36 and 0.38%, respectively, for conventional, organic and biodynamic systems. Using of all farming systems (conventional, organic and biodynamic) enhanced total nitrogen, total phosphorus and total potassium in different proportions compared to the beginning of the experiment. The average values of the total nitrogen, total phosphorus and total potassium were 0.13, 0.17 and 0.20, 0.31, 0.23 and 0.18 and 0.09, 0.24 and 0.24%, respectively, for conventional, organic and biodynamic systems. These results agreed with those obtained by Krause et al.^3^ whose found with the use of biodynamic farming system improved soil quality compared to other systems.

### Water consumption

**Fig. 2:**
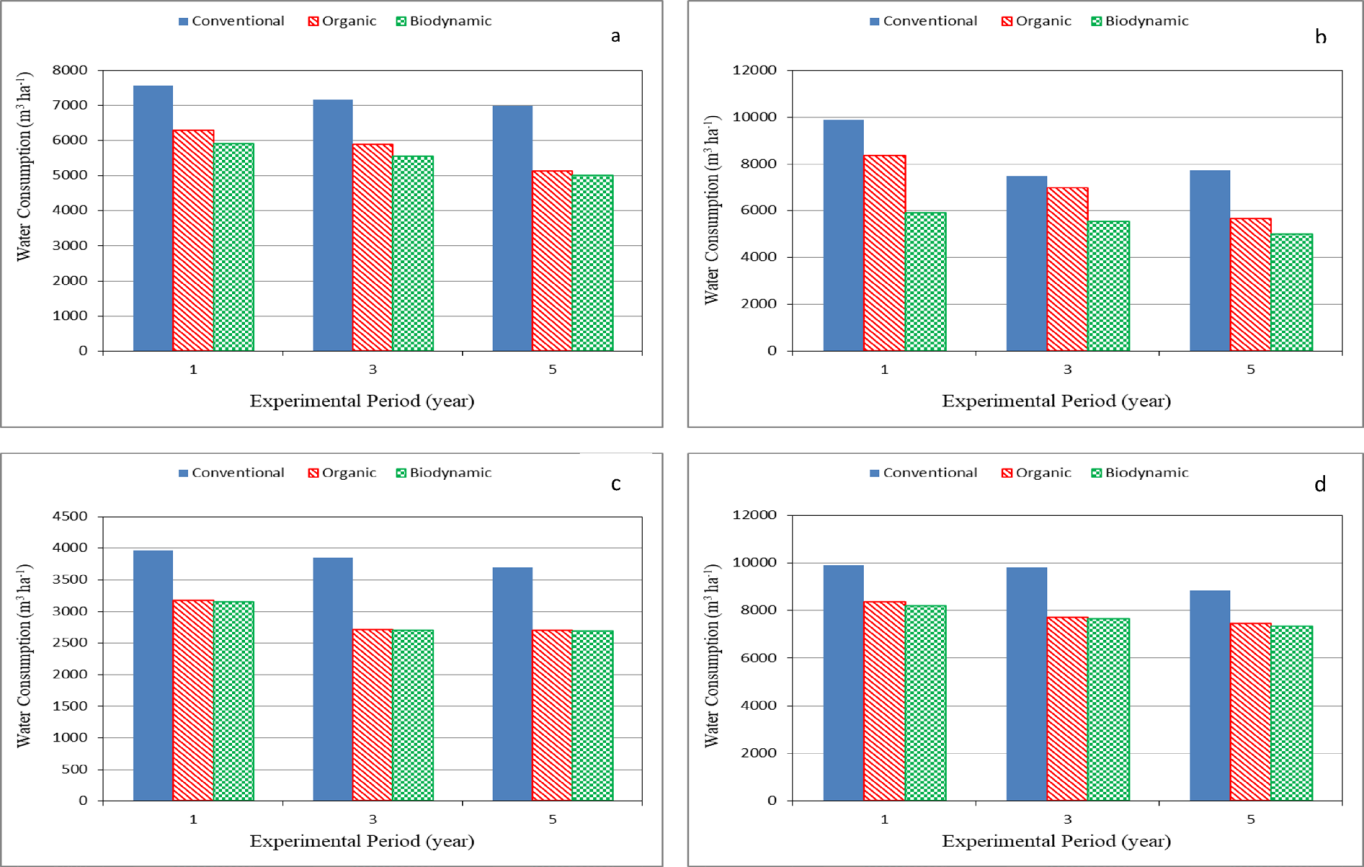
Water consumption for different crops grown in the different farming systems. (**a**) Maize (**b**) Tomato (**c**) Faba bean (**d**) Potato.

Figure 2 shows the water consumption for different crops (maize, tomato, faba bean and potato) grown under different farming systems (conventional, organic and biodynamic) during experimental period. The results indicate that the water consumption for different crops increased during the experimental period for conventional farming system more than the other farming systems (organic and biodynamic), because the use of biofertilizers increases the water holding capacity. It could be seen that the water consumption ranged from 6989 to 7560, 5136 to 5882 and 5002 to 5904 m^3^ ha^− 1^ for conventional, organic and biodynamic systems, respectively, for maize crop. For tomato crop, the water consumption ranged from 7488 to 9888, 5640 to 8352 and 5508 to 8184 m^3^ ha^− 1^ for conventional, organic and biodynamic systems, respectively. For faba bean crop, the water consumption ranged from 3696 to 3960, 2700 to 3168 and 2688 to 3144 m^3^ ha^− 1^ for conventional, organic and biodynamic systems, respectively. For potato crop, the water consumption ranged from 8832 to 9888, 7440 to 8352 and 7320 to 8184 m^3^ ha^− 1^ for conventional, organic and biodynamic systems, respectively.

Using of biodynamic and organic farming systems saved water compared to the conventional farming system. The rates of saving water by using biodynamic and organic farming systems were 24.23 and 20.29, 18.17 and 16.50, 25.84 and 25.43 and 18.85 and 17.59%, respectively, for maize, tomato, faba bean and potato crops.

#### Crop yield

**Fig. 3:**
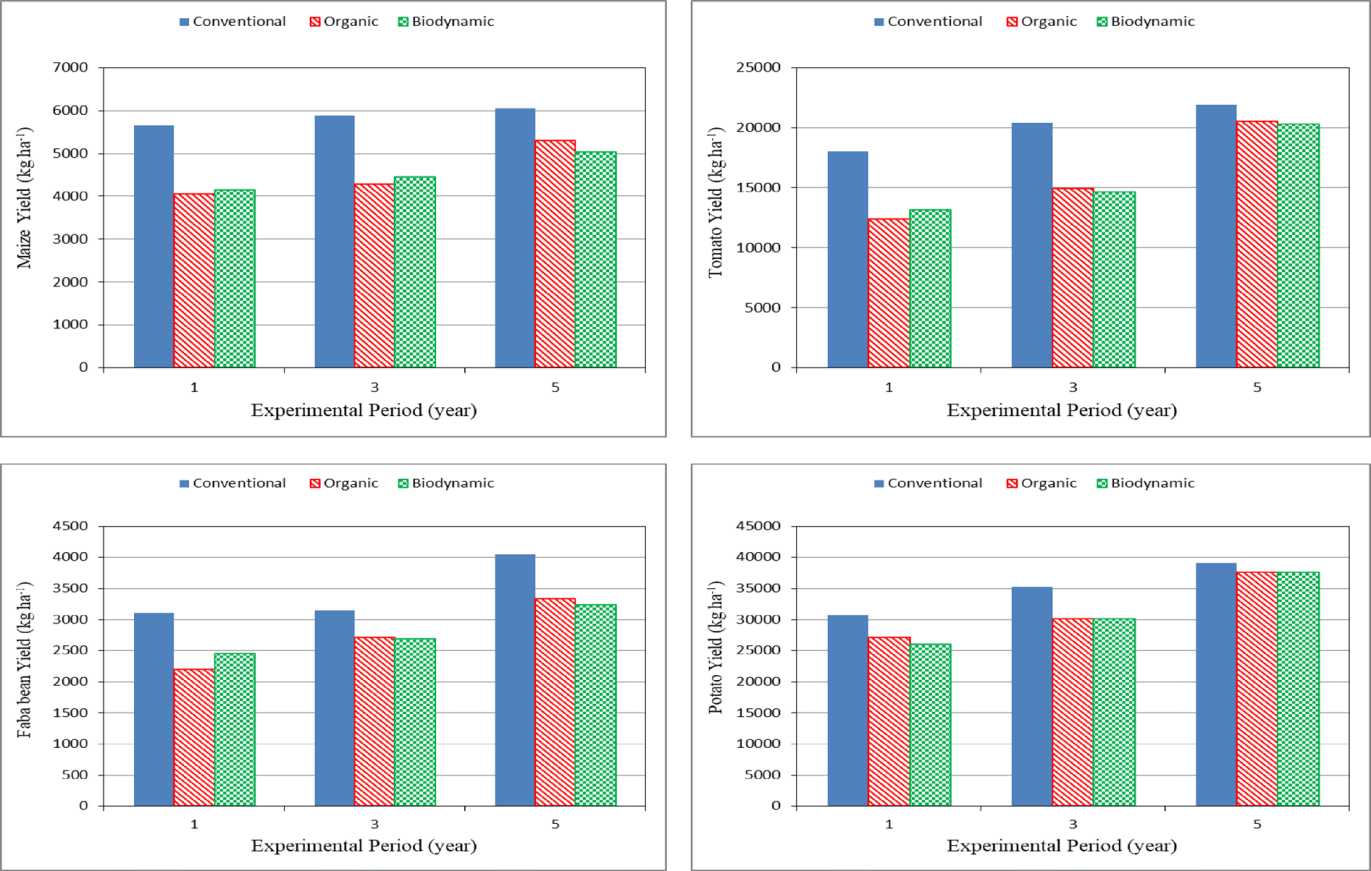
Yield for different crops grown in the different farming systems.

Figure 3 shows the yield of different crops (maize, tomato, faba bean and potato) grown for different farming systems (conventional, organic and biodynamic) at the end of experimental period. The results indicate that the crops yield increased during the experimental period for conventional farming system more than the other farming systems (organic and biodynamic). This is due to the use of chemical fertilizers for different crops at the beginning of agricultural seasons led to an increase in yield compared to other farming systems.

It could be seen that the maize yield ranged from 5650 to 6043, 4051 to 5304 and 4140 to 5040 kg ha^− 1^ for conventional, organic and biodynamic systems, respectively. The tomato yield ranged from 18,034 to 21,888, 12,384 to 20,544 and 13,128 to 20,280 kg ha^− 1^ for conventional, organic and biodynamic systems, respectively. The faba bean yield ranged from 3103 to 4049, 2196 to 3331 and 2455 to 3240 kg ha^− 1^ for conventional, organic and biodynamic systems, respectively. For potato crop, the potato yield ranged from 30,720 to 39,072, 27,120 to 37,560 and 25,980 to 37,632 kg ha^− 1^ for conventional, organic and biodynamic systems, respectively.

The results obtained clarified that the use of chemical fertilizers for different crops (maize, tomato, faba bean and potato) led to an increase in yield compared to other systems. These results agreed with those obtained by Dhillon et al.^41^.

Due to the improvement of soil properties under the use of the different farming systems (conventional, organic and biodynamic) during experimental period, it gave the highest yield for the fifth season compared to other seasons. The maize yield increased by 6.97, 30.92 and 21.79% for conventional, organic and biodynamic, respectively, after five year. The tomato yield increased by 21.37, 65.89 and 54.48% for conventional, organic and biodynamic, respectively, while, the faba bean yield increased by 30.47, 51.69 and 31.96% for conventional, organic and biodynamic, respectively, and the potato yield increased by 27.19, 38.50 and 44.85% for conventional, organic and biodynamic, respectively. The obtained results are in agreement with the findings by Hirte et al.^42^, Timsina^43^ and Krause et al.^3^.

### Water use efficiency

**Fig. 4:**
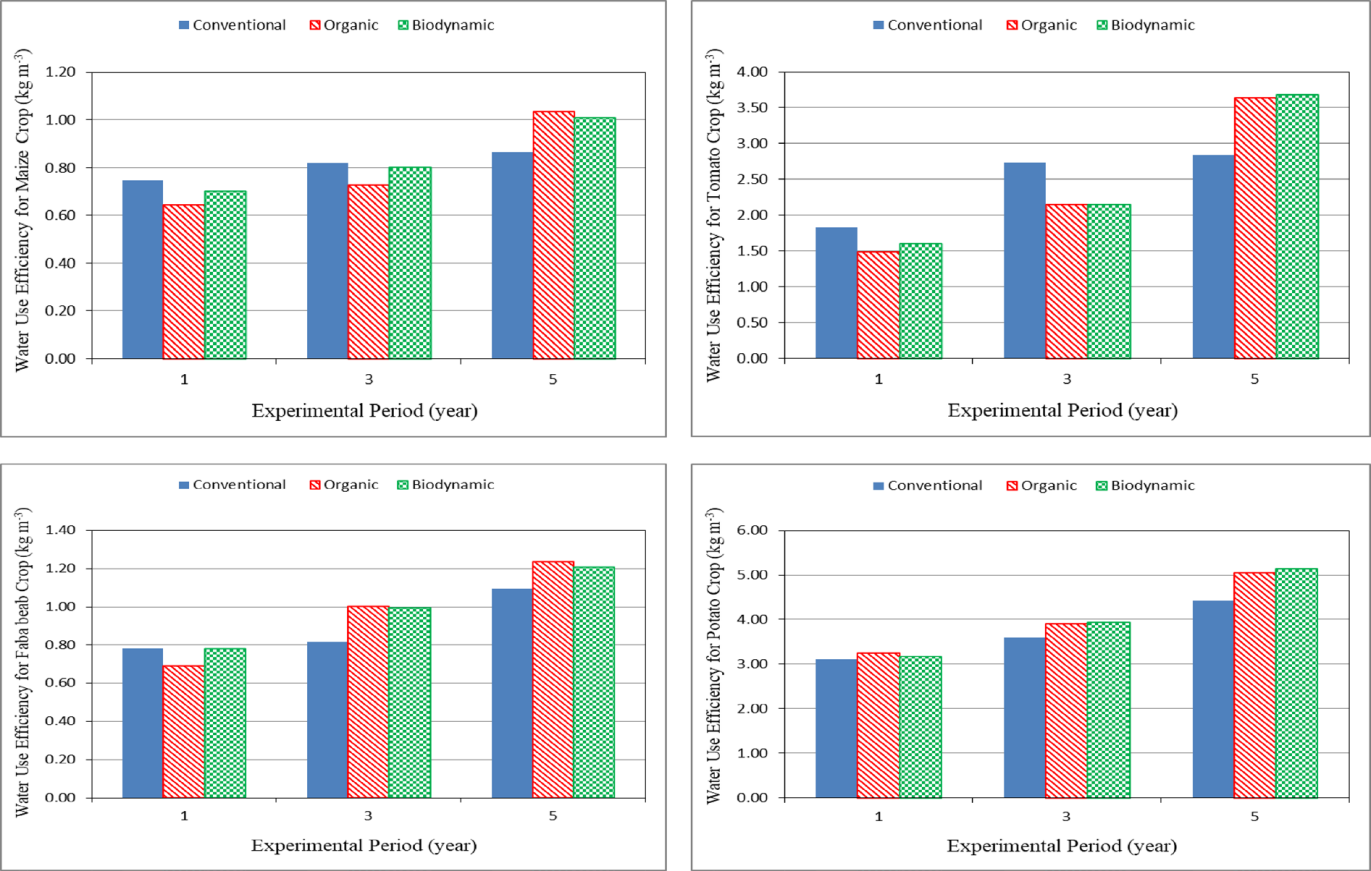
Water use efficiency for different crops grown in the different farming systems.

Figure 4 shows the water use efficiency (WUE) for different crops (maize, tomato, faba bean and potato) grown for different farming systems (conventional, organic and biodynamic) at the end of experimental period. The results indicate that the water use efficiency increases with increasing the experimental season for different farming systems (conventional, organic and biodynamic). It could be seen that the water use efficiency for maize crop significantly increased from 0.75 to 0.86, 0.64 to 1.03 and 0.70 to 1.01 kg m^− 3^, when the experimental season increased from 1 to 5 years for conventional, organic and biodynamic systems, respectively. The water use efficiency for tomato crop significantly increased from 1.82 to 2.84, 1.48 to 3.64 and 1.60 to 3.68 kg m^− 3^, when the experimental season increased from 1 to 5 years for conventional, organic and biodynamic systems, respectively. The water use efficiency for faba bean crop significantly increased from 0.78 to 1.10, 0.69 to 1.23 and 0.78 to 1.21 kg m^− 3^, when the experimental season increased from 1 to 5 years for conventional, organic and biodynamic systems, respectively. For potato crop, the water use efficiency significantly increased from 3.11 to 4.42, 3.25 to 5.05 and 3.17 to 5.14 kg m^− 3^, when the experimental season increased from 1 to 5 years for conventional, organic and biodynamic systems, respectively.

The results indicate that the water use efficiency values for different crops during the experimental period for organic and biodynamic farming systems were higher than that of the conventional farming system, because the use of biofertilizers enhanced water use efficiency. It could be seen that, the water use efficiency for organic and biodynamic farming systems increased by 0.19 and 0.17, 0.28 and 0.30, 0.13 and 0.10 and 0.14 and 0.16%, respectively, compared to the conventional farming system at the 5th year.

### Effect of farming systems on soil carbon sequestration

**Fig. 5:**
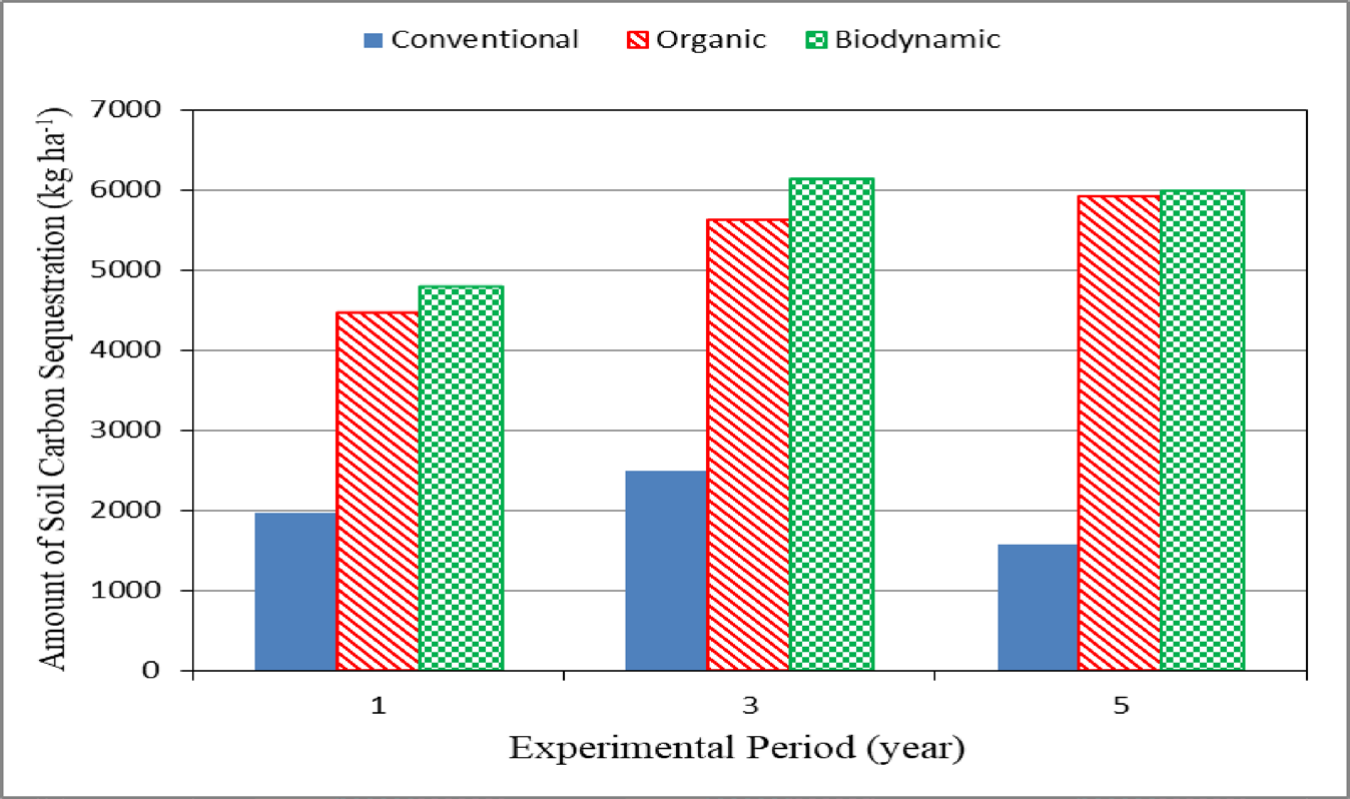
Amount of soil carbon sequestration for different farming systems.

Figure 5 shows the amount of soil carbon sequestration (SCS) for different farming systems (conventional, organic and biodynamic) during experimental period. The results indicate that the amount of soil carbon sequestration (SCS) for conventional system was lower than those of organic and biodynamic systems during experimental period. It could be seen that the amount of soil carbon sequestration (SCS) values were 1980.16, 2505.88 and 1581.08, 4456.13, 5617.48 and 5919.72 and 4782.82, 6132.38 and 5986.25 kg ha^− 1^ for conventional, organic and biodynamic systems, respectively, after 1, 3 and 5 years of agriculture. These results agreed with those obtained by Berner et al.^44^ whose designed a factorial field experiment on reduced soil tillage, manure management, and biodynamic preparations. Consequently, we assume an improved organic input quality after long manure composting under aerobic conditions to be the main factor for enhanced soil quality and increasing SOC contents. The amount of SCS increased through the 3rd year of agriculture and then decreased. This could be explained that the soil density is one of the main factors affecting the SOC in soil increased with time, which the density decreased due the increasing of organic matter in the soil which will decrease the SOC by the time.

### Amount of CO_2_ emission reduction

**Fig. 6:**
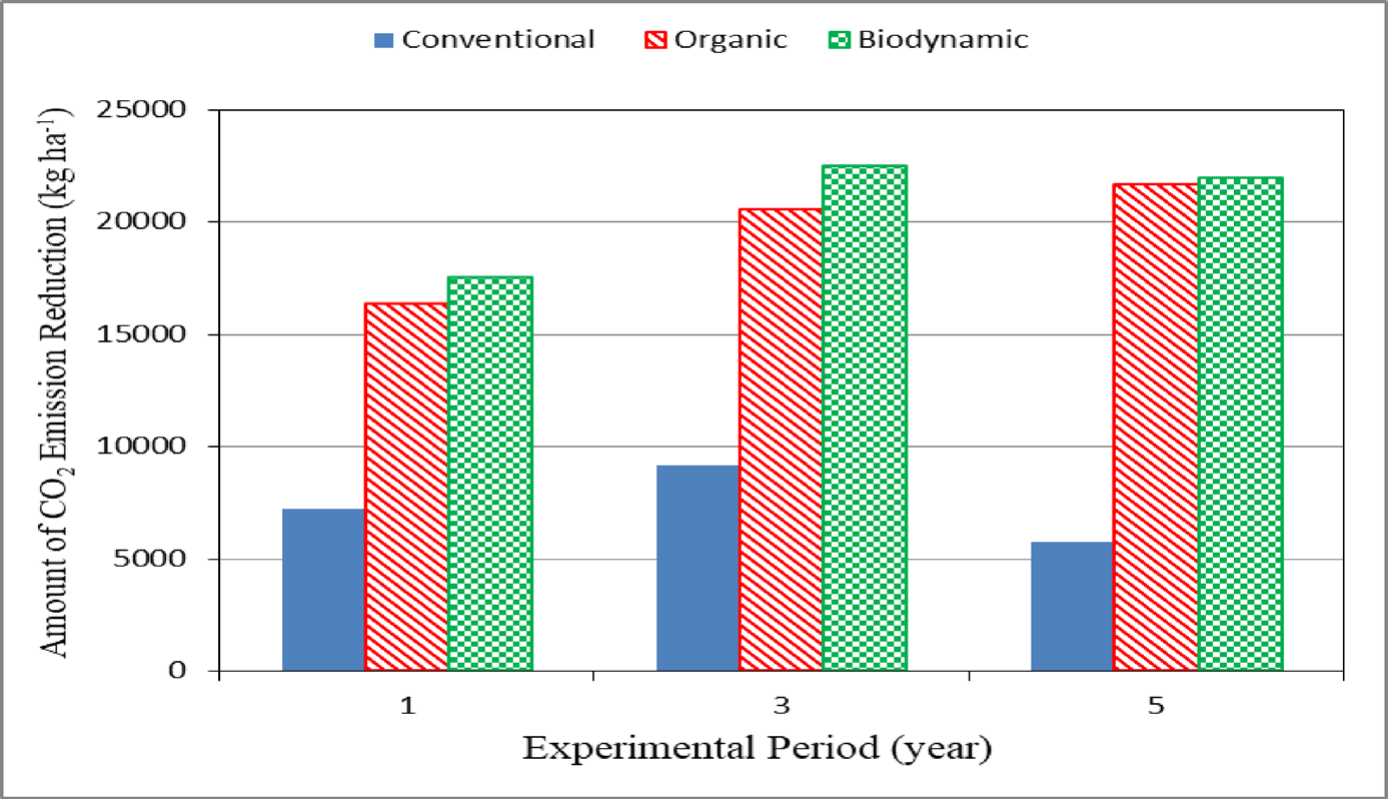
Amount of mitigation of CO_2_ emission for different farming systems.

Figure 6 shows the amount of CO_2_ emission reduction for different farming systems (conventional, organic and biodynamic) during experimental period. The results indicate that the amount of CO_2_ emission reduction for organic and biodynamic systems were higher than those of conventional system during experimental period. It could be seen that the amount of CO_2_ emission reduction was 7255.31, 9181.53 and 5793.06 and 16327.25, 20582.46 and 21689.85 and 17524.25, 22469.03 and 21933.65 kg ha^− 1^ for conventional, organic and biodynamic systems, respectively, after 1, 3 and 5 years of agriculture. These results agreed with those obtained by Shin et al.^45^.

### Cost estimation

Table 7 shows the total cost, production cost, total return, net return, Carbon profit and total net profit different crops (maize, tomato, faba bean and potato) grown for different farming systems (conventional, organic and biodynamic) at the end of experimental period. The results indicate that the total cost of maize crop ranged from 8923 to 21,859, 8146 to 18,070 and 8441 to 19,013 EGP ha^− 1^ for conventional, organic and biodynamic systems, respectively. The total cost of tomato crop ranged from 42,310 to 65,126, 38,942 to 63,283 and 39,108 to 62,904 EGP ha^− 1^ for conventional, organic and biodynamic systems, respectively. The total cost of faba bean crop ranged from 5755 to 16,565, 6826 to 16,745 and 7217 to 17,933 EGP ha^− 1^ for conventional, organic and biodynamic systems, respectively. The total cost of potato crop ranged from 18,890 to 77,326, 17,688 to 71,940 and 17,825 to 72,773 EGP ha^− 1^ for conventional, organic and biodynamic systems, respectively.

**Table 7:** Cost Estimation of different crops grown for different farming systems.

Item	Crop	Farming system								
		Conventional			Organic			Biodynamic		
		1st year	3rd year	5th year	1st year	3rd year	5th year	1st year	3rd year	5th year
		Cost, EGP ha^− 1^								
Total cost, EGP ha^− 1^	Maize	8923^c^	13,606^f^	21,859^i^	8146^a^	10,490^d^	18,070^g^	8441^b^	11,285^e^	19,013^h^
	Tomato	42,310^c^	55,450^f^	65,126^i^	38,942^a^	51,156^e^	63,283^h^	39,108^b^	51,012^d^	62,904^g^
	Faba bean	5755^a^	10,951^d^	16,565^g^	6826^b^	12,842^e^	16,745^h^	7217^c^	13,476^f^	17,933^i^
	Potato	18,890^b^	53,724^d^	77,326^g^	17,688^a^	50,112^c^	71,940^e^	17,825^a^	50,496^c^	72,773^f^
Production cost, EGP kg^− 1^	Maize	3.3^a^	4.0^b^	4.3^c^	4.3^c^	5.1^d^	5.8^e^	4.3^c^	5.1^d^	5.8^e^
	Tomato	3.4^a^	4.0^b^	4.3^c^	4.3^c^	5.1^d^	5.8^e^	4.3^c^	5.1^d^	5.8^e^
	Faba bean	7.0^a^	8.8^c^	12.5^e^	8.4^b^	9.1^d^	14.5^f^	8.4^b^	9.1^d^	14.5^f^
	Potato	1.5^a^	2.1^c^	3.2^e^	1.8^b^	2.8^d^	4.1^f^	1.8^b^	2.8^d^	4.1^f^
Total return, EGP ha^− 1^	Maize	18,643.7^b^	23,520.0^e^	25,683.6^f^	17217.6^a^	21,848.4^c^	30,498.0^h^	17,595.0^a^	22,644.0^d^	28,980.0^g^
	Tomato	61,314.2^c^	81,792.0^e^	93,024.0^f^	52,632.0^a^	76,132.8^d^	118,128.0^h^	55,794.0^b^	74,688.5^d^	116,610.0^g^
	Faba bean	21,722.4^c^	27,709.4^e^	50,610.0^h^	18,446.4^a^	24,701.0^d^	48,302.4^g^	20,623.7^b^	24,460.8^d^	46,980.0^f^
	Potato	46,080.0^a^	74,088.0^c^	125,030.4^e^	47,460.0^b^	82,830.0^d^	153,996.0^f^	45,465.0^a^	82,698.0^d^	154,291.2^f^
Net return, EGP ha^− 1^	Maize	9720.5^c^	9914.4^d^	3824.4^a^	9072.0^b^	11,358.0^f^	12,428.4^g^	9154.2^b^	11,359.2^f^	9967.2^d^
	Tomato	19,004.6^c^	26,342.4^f^	27,897.6^g^	13,689.6^a^	24,976.8^e^	54,844.8^i^	16,686.0^b^	23,676.5^d^	53,706.0^h^
	Faba bean	15,967.2^d^	16,758.2^e^	34,045.2^h^	11,620.8^b^	11,858.6^b^	31,557.6^g^	13,406.9^c^	10,984.8^a^	29,047.2^f^
	Potato	27,189.6^b^	20,364.0^a^	47,704.8^e^	29,772.0^c^	32,718.0^d^	82,056.0^f^	27,640.2^b^	32,202.0^d^	81,518.4^f^
Carbon profit, EGP ha^− 1^		4220.9^a^	5341.4^b^	3370.2^a^	9498.5^c^	11,974.1^d^	12,618.3^d^	10,194.9^c^	13,071.6^de^	12,760.1^d^
Total net profit, EGP ha^− 1^	Maize	13,941.3^b^	15,255.8^c^	7194.6^a^	18,570.5^d^	23,332.1^e^	25,046.7^ef^	19,349.1^d^	24,430.8^e^	22,727.3^e^
	Tomato	23,225.5^a^	31,683.8^c^	31,267.8^c^	23,188.1^a^	36,950.9^d^	67,463.1^e^	26,880.9^b^	36,748.1^d^	66,466.1^e^
	Faba bean	20,188.1^a^	22,099.7^c^	37,415.4^e^	21,119.3^b^	23,832.7^d^	44,175.9^g^	23,601.8^d^	24,056.4^d^	41,807.3^f^
	Potato	31,410.5^b^	25,705.4^a^	51,075.0^g^	39,270.5^d^	44,692.1^f^	94,674.3^h^	37,835.1^c^	45,273.6^f^	94,278.5^h^

$ = 48.48 EGP.

Means on the same row with different superscripts are significantly different (*p* < 0.05)

The results also indicate that the total return of maize crop ranged from 18643.7 to 25,683.6, 17217.6 to 30,498.0 and 175,955.0 to 28,980.0 EGP ha^− 1^ for conventional, organic and biodynamic systems, respectively. The total return of tomato crop ranged from 61,314.2 to 93,024.0, 52,632.0 to 118,128.0 and 55,794.0 to 116,610.0 EGP ha^− 1^ for conventional, organic and biodynamic systems, respectively. The total return of faba bean crop ranged from 21,722.4 to 50,610.0, 18,446.4 to 48,302.4 and 20,623.7 to 46,980.0 EGP ha^− 1^ for conventional, organic and biodynamic systems, respectively. The total return of potato crop ranged from 46080.0 to 125030.4, 47460.0 to 153996.0 and 45465.0 to 154291.2 EGP ha^− 1^ for conventional, organic and biodynamic systems, respectively.

The carbon profit was 4220.9, 5341.4 and 3370.2, 9498.5, 11974.1 and 12,618.3 and 10,194.9, 13071.6 and 12,760.1 EGP ha^− 1^ for conventional, organic and biodynamic systems, respectively, after 1, 3 and 5 years of agriculture. The carbon profit for organic and biodynamic systems was higher than those of conventional system during experimental period. The highest value of carbon profit (13071.6 EGP ha^− 1^) was found with the biodynamic system, while, the lowest value of carbon profit (3370.2 EGP ha^− 1^) was found with the conventional system.

The total net profit of maize crop was 13,941.3, 15,255.8 and 7194.6, 18,570.5, 23,332.1 and 25,046.7 and 19,349.1, 24,430.8 and 22,727.3 EGP ha^− 1^ for the first, third and fifth agricultural season, respectively, for conventional, organic and biodynamic systems. The total net profit of tomato crop was 23,225.5, 31,683.8 and 31,267.8, 23,188.1, 36,950.9 and 67,463.1 and 26,880.9, 36748.1 and 66,466.1 EGP ha^− 1^ for the first, third and fifth agricultural season, respectively, for conventional, organic and biodynamic systems. The total net profit of faba bean crop was 20,188.1, 22,099.7 and 37,415.4, 21,119.3, 23,832.7 and 44,175.9 and 23,601.8, 240,556.4 and 41,807.3 EGP ha^− 1^ for the first, third and fifth agricultural season, respectively, for conventional, organic and biodynamic systems. The total net profit of potato crop was 31,410.4, 25,705.4 and 51,075.0, 39,270.5, 44,692.1 and 94,674.3 and 37,835.1, 45273.6 and 94,278.5 EGP ha^− 1^ for the first, third and fifth agricultural season, respectively, for conventional, organic and biodynamic systems. Based on the foregoing, the result indicated that the ability of the biodynamic farming system to increase the total net profit despite the stability of the selling price of the final products under different farming systems. These results agreed with those obtained by Sinha et al.^46^.

## Conclusion

The experiment was carried out successively to investigate the effect of the agricultural practice systems on the soil properties, yield, water consumption, CO_2_ emission and cost of some agricultural crops. It is concluded that the agricultural practices for different farming systems enhanced the soil properties. The yield crop ranged from 4051 to 6043, 12,384 to 21,888, 2196 to 4049 and 27,120 to 39,072 kg ha^− 1^ for maize, tomato, faba bean and potato crops, respectively, for all treatments under study. The water use efficiency for organic and biodynamic farming systems more increased by 0.19 and 0.17, 0.28 and 0.30, 0.13 and 0.10 and 0.14 and 0.16%, respectively, for conventional farming system in the fifth season. The increasing present of soil carbon sequestration were 55.56 and 55.39, 73.29 and 58.59 and 59.14 and 73.60% for conventional, organic and biodynamic systems, respectively after 3 and 5 years of agriculture. The highest value of the amount of soil carbon sequestration (6132.38 kg ha^− 1^) was found with organic system. The highest value of carbon profit (13071.6 EGP ha^− 1^) was found with the biodynamic system. The highest values of total net profit were 25046.7, 67463.1, 44175.9 and 94674.3 kg ha^− 1^ for maize, tomato, faba bean and potato crops, respectively, were found with the organic farming system after 5 agricultural years. Further studies are recommended to apply wide range of biodynamics farming levels in fruits and vegetable crops.

## Data Availability

The datasets used and/or analyzed during the current study available from the corresponding author on reasonable request.

## List of symbols

$Am_{{{\mathrm{CO}}_{2} }}$: Amount of mitigation of CO_2_ emission
BD: Bulk density
C/N: Carbon nitrogen ratio
d: Soil depth
EC: Electrical conductivity
CF: Conversion factor of CO_2_ emission from carbon
Es: Drip irrigation system efficiency
IWR: Irrigation water requirements
L_*f*_: Leaching factor
Mp: Market prices of CO_2_ offsets
MC: Moisture content
NR: Net return
PCO_2_: Profit analysis for mitigation of CO_2_ emission
SOM: Soil organic matter
SOC: Soil organic carbon
SCS: Soil carbon sequestration
TC: Total costs
TR: Total return
WHC: Water holding capacity
*W*_*i*_: Weight of sample before drying
*W*_*s*_: Weight of sample after drying
WUE: Water use efficiency
θ_FC_: Soil moisture content at field capacity
θ_v_: Soil moisture content before irrigation
